# Associations between sleep disorders and anxiety in patients with tinnitus: A cross-sectional study

**DOI:** 10.3389/fpsyg.2022.963148

**Published:** 2022-08-05

**Authors:** Shenglei Wang, Xudong Cha, Fengzhen Li, Tengfei Li, Tianyu Wang, Wenwen Wang, Zhengqing Zhao, Xiaofei Ye, Caiquan Liang, Yue Deng, Huanhai Liu

**Affiliations:** ^1^Department of Otolaryngology, Changzheng Hospital, Naval Medical University (Second Military Medical University), Shanghai, China; ^2^Department of Neurology, Changzheng Hospital, Naval Medical University (Second Military Medical University), Shanghai, China; ^3^Department of Health Statistics, Naval Medical University (Second Military Medical University), Shanghai, China

**Keywords:** tinnitus, tinnitus severity, anxiety, sleep disorders, mediating effect

## Abstract

**Objective:**

To investigate the characteristics of sleep disorders and anxiety in patients with tinnitus, their influencing factors, and the role of sleep disorders as mediators.

**Methods:**

The general conditions and disease characteristics of 393 patients with tinnitus presented to the Changzheng Hospital of the Naval Medical University from 2018 to 2021 were collected. All patients accepted questionnaires such as Tinnitus Handicap Inventory (THI), Pittsburgh Sleep Quality Index (PSQI) and Self-rating Anxiety Scale (SAS), and then the characteristics and the influencing factors of sleep disorders and anxiety were analyzed.

**Results:**

Among the 393 tinnitus patients, 213 cases (54.19%) were diagnosed with sleep disorders, and 78 cases (19.85%) were diagnosed with anxiety, including 25 men (32.1%) and 53 women (67.9%). Binary regression showed that gender, hearing loss, tinnitus severity, and sleep disorders severity were positively associated with anxiety. Multiple logistic regression analysis showed that female gender (OR = 2.526, *P* = 0.008), hearing loss (OR = 2.901, *P* = 0.003, tinnitus severity (OR = 1.863, *P* = 0.003) and sleep disorders (OR = 2.510, *P* = 0.001) were the independent risk factors of anxiety. The mediating effect of sleep disorders between tinnitus severity and anxiety accounted for 27.88% of the total effect size.

**Conclusion:**

Females patients with hearing loss, moderate to severe tinnitus, and sleep disorders were at greater risk for anxiety, with sleep disorders partially mediating the anxiety associated with tinnitus.

## Introduction

Tinnitus is the sensation of sound in the ear or head without an external acoustic source. Tinnitus is a common audiological disorder that affects 10.1% of the adult population worldwide (Chang et al., 2019). The sounds are always considered uncomfortably or unpleasantly loud, and even 0.5–1.6% of the patients consider it severely annoying to affect the normal life (Baguley et al., 2013; Tyler et al., 2014).

The causes of tinnitus are complex, occupational or recreational noise exposure are clearly associated with tinnitus, and tinnitus can develop as a result of increased life stress (Kim et al., 2015; Rhee et al., 2020; Szczepek and Mazurek, 2021). Age-related hearing loss and hyperacusis have been linked to tinnitus, which was found to be more common and severe as people aged (Gallus et al., 2015). In China, the prevalence increased sharply after the age of 50 and plateaued at around 32% among individuals over 70 (Zhang et al., 2021). Heart illness, head and neck injuries, the use of steroid medicines and other diseases may hasten or contribute to the development of tinnitus in the elderly (Jafari et al., 2019). Current models consider cochlear damage as the basis of tinnitus. Stochastic resonance is assumed to lead to neuronal hyperactivity and tinnitus (Krauss et al., 2016, 2019).

Various psychological or psychosomatic symptoms, notably anxiety, depression, and sleep disorders, have been linked to tinnitus (Izuhara et al., 2013). Sleep disorders are the most common symptom associated with tinnitus, with an incidence of up to 60% (Aazh et al., 2019). Based on previous studies, sleep disorders can exacerbate the distress of tinnitus and cause daytime fatigue and drowsiness (Li et al., 2022). Anxiety is often manifested as chronic excessive worry, fear, and avoidance behaviors, which can seriously affect patients' quality of daily life (Craske and Stein, 2016). Studies have shown that people with tinnitus score significantly higher than the general population on anxiety and depression scales (Pattyn et al., 2016). The prevalence of anxiety in tinnitus patients was 24–42.1%, while the general population prevalence was 7.3% (Craske and Stein, 2016; Aazh and Moore, 2017; Li et al., 2022). Anxiety symptoms are more likely to cause depression than vice versa. Meanwhile, the influence of tinnitus on depression was proved to be mainly mediated by sleep disorders, hyperacusis and anxiety, though the effect of sleep disorders on anxiety was not described (Aazh and Moore, 2017). Previous research has established that sleep disorders can promote the occurrence of anxiety and may raise the risk of emotional distress in tinnitus patients (Richter et al., 2021). Sleep disorders worsen the severity of most symptoms of anxiety. However, the mechanism was unclear and may be related to impaired emotional regulation, cognitive impairment, and circadian rhythm disorder caused by insufficient sleep (Chance Nicholson and Pfeiffer, 2021).

In China, tinnitus is currently treated by tinnitus habituation therapy and sound therapy. Besides, psychological disorders in tinnitus patients are receiving increasing attention. Adequate diagnosis and treatment of psychiatric disorders associated with tinnitus can significantly improve patients' quality of life (Pinto et al., 2014; Pattyn et al., 2016). However, the relationship between sleep disorders and anxiety in tinnitus patients has not been well-studied, especially for anxiety as an outcome. The aims of this study were to assess the prevalence of sleep disorders and anxiety, and identify their risk factors. We used mediation analysis to examine whether sleep disorders mediate the relationship between tinnitus severity and anxiety. The clinical characteristics, quality of sleep, anxiety situation, and tinnitus severity were investigated in our study.

## Methods

### Study design and participants

To assess the prevalence of sleep disorders and anxiety in patients with tinnitus in one clinic, we conducted a descriptive, cross-sectional survey study.

This study included 393 patients with tinnitus as a primary complaint who attended the Department of Otolaryngology outpatient clinic at Shanghai Changzheng Hospital from September 2018 to February 2021. Participants completed audiological tests and Chinese version questionnaires such as the Tinnitus Handicap Inventory (THI), Pittsburgh Sleep Quality Index (PSQI) and Self-rating Anxiety Scale (SAS).

Inclusion criteria: tinnitus as the primary complaint; written informed consent signed by patients and their families; agreement to participate in the survey. Patients with the following conditions were excluded: age < 18 or > 85 years; objective tinnitus; carotid body tumor; acute or chronic external or media otitis; middle ear cholesteatoma; otosclerosis; Meniere's disease; ear surgery; severe cardiovascular and cerebrovascular diseases; severe mental diseases or undergoing anti-anxiety or depression treatment. Patients who could not complete the questionnaires or cooperate with audiological and tinnitus tests were excluded.

The study protocol was approved by the Medical Ethics Committee of the Shanghai Changzheng Hospital (2018SLYS1). In addition, all participants or their families signed written informed consent.

### Sample size

G^*^Power 3.1.9.7 program was used to calculate the sample size, with a linear multiple regression model. The statistical significance level was set at α = 0.05, the effect size of 0.1, the statistical power (1-β) of 0.90, and total predictor numbers of 11. Theoretically, a minimum sample size of 108 was calculated.

### Questionnaires and data acquisition

All patients' tinnitus histories and general information were meticulously evaluated and recorded. Concurrently, all patients were administered the questionnaires outlined below, pure-tone audiometry, and the psychoacoustic measurements of tinnitus, including tinnitus pitch matching and tinnitus loudness matching. All the tinnitus data were measured and calculated by the same experienced audiologist.

Tinnitus loudness, frequency and sound types were detected based on the patients' tinnitus side. Then factors associated with tinnitus were established, including gender, age, localization (left, right, and both ears), tinnitus duration (acute tinnitus as tinnitus duration < 3 months, subacute tinnitus as 3–6 months duration, and chronic tinnitus as duration > 6 months), tinnitus frequency (≤ 500 Hz, 500–3,000 Hz, and ≥ 3,000 Hz), loudness (≤ 25 dB, ≥ 26 dB), tinnitus sound types (pure tones, and compound tones), and hearing status (normal and impairment). Hearing impairment was determined by calculating the mean pure-tone air conduction hearing threshold at 500, 1,000, and 2,000 Hz. Hearing threshold means ≤ 25 dB were normal, and ≥ 26 dB were assessed as having a hearing loss.

#### Tinnitus handicap inventory

The Tinnitus Handicap Inventory (THI) quantified the impact of tinnitus on patients' daily life and measured its severity. The Chinese-Mandarin version of the THI has a high test-retest (*r* = 0.98) and internal consistency reliability (α = 0.93) (Meng et al., 2012). The THI scale comprises three subscales, incorporating functional, emotional and catastrophic subscales, and it contains 25 items for a total of 100 points. Tinnitus severity was classified into five levels based on the THI score: 0–16 as slight, 18–36 as mild, 38–56 as moderate, 58–76 as severe, and 78–100 as catastrophic (Newman et al., 1996).

#### Self-rating anxiety scale

The Self-rating Anxiety Scale, proposed by Zung (1971), was used to evaluate the severity of patients' anxiety over the past week. The SAS scale consists of 20 items, and each item is scored at four levels. One point means “no or little time,” and four points mean “most or all time” (Zung, 1971). The SAS scale is widely used in China due to its validity and reliability, with internal consistency and test-recovery reliability values of 0.93 and 0.77 (Shi et al., 2019). The total score of each item was multiplied by 1.25, and the integral part is SAS standard score. A SAS score of ≥ 50 suggests anxiety symptoms or the anxiety state, with 50–59 representing mild anxiety, 60–69 representing moderate anxiety, and ≥ 70 representing severe anxiety.

#### Pittsburgh sleep quality index

The Pittsburgh Sleep Quality Index (PSQI) exhibits a high overall scale and test-retest reliability in China (α = 0.82–0.83, *r* = 0.77–0.85). This study used it to access patients' sleep status (Tsai et al., 2005). The PSQI was divided into seven parts, including subjective sleep quality, sleep latency, sleep duration, sleep efficiency, sleep disturbances, use of sleeping pills, and daytime dysfunction (Buysse et al., 1989). The total score ranges from 0 to 21, with higher scores indicating poorer sleep quality. Patients with a score ≥ 5 were considered to have a sleep disorder, with 5–10 being classified as mild, 11–15 as moderate, and 16–21 as severe.

#### Statistical analysis

IBM SPSS 25.0 was used to analyze and calculate the included patients' baseline data, audiological measurement results, THI, SAS and PSQI scores. The non-normally distributed variables were presented by medians and interquartile ranges (IQR). Means ± standard deviations (SD) were calculated for variables with a normal distribution. Frequencies and percentages are used to calculate the statistics for the distribution of different groups. The Mann-Whitney U test was used for the comparison of the variables that did not conform to the normal distribution between the two groups (age, THI scores, SAS scores and PSQI scores). Multiple groups were compared by the Kruskal-Wallis H test simultaneously. The whole data was randomly split into the training set and validation set with a ratio of 3:7. The training set was used for selecting potential covariates by binary logistic regression analysis with anxiety or sleep disorders performed as dependent variables. The multiple logistic regression model was tested using the data from the validation set. The false discovery rate (FDR) approach was used to correct the *p*-value for multiple tests. Variables were chosen for a multiple logistic regression model only if its FDR corrected *p* < 0.15.

The SPSS AMOS 24.0 and Bootstrap methods were used to analyze and verify the mediating effect between THI and SAS, with PSQI serving as the mediator variable. The mediation analysis calculated the regression coefficient (β) between the variables to assess their direct and indirect effects on the dependent variable. By multiplying the regression coefficients between the independent variable and the mediating variable and the regression coefficients between the mediating variable and the dependent variable, indirect effects were calculated. The direct influence of the independent variable on the dependent variable was called the direct effect, and the total effect was the sum of the direct and indirect effects. If the 95 percent confidence interval (95% CI) of Bootstrap did not contain zero, the relationship was significant. *P* < 0.05 was regarded as statistically significant in all statistical analyses, except for the FDR level of the simple logistic regression to screen for potential covariates.

## Results

### General situation, tinnitus characteristics and psychopathological factors of patients

Our study included 393 patients, 208 of whom were male and 185 were female. Patients' ages ranged from 18 to 85 years, with an average of 52.80 ± 14.67 years. Tinnitus duration was < 3 months in 122 cases, 3–6 months in 36 cases, and > 6 months in 200 cases. There were 118 cases of tinnitus localization in the left ear, 93 cases in the right ear and 182 cases in both ears. Tinnitus frequency occurred at ≤ 500 Hz in 35.11% of patients; 500–3,000 Hz in 8.14%; and mostly concentrated in ≥ 3,000 Hz range as 55.98%. In 216 patients (54.96%), tinnitus sound types were reported as compound tones, and the mean tinnitus loudness was 46.37 ± 18.36 dB. Hearing loss was reported as a pure-tone audiometry threshold ≥ 26 dB in 191 patients, accounting for 48.60%. The average THI score was 31.28 ± 17.18, and 277 patients (70.4%) had mild to moderate tinnitus. The mean SAS score of 393 patients was 45.14 ± 6.78, and anxiety symptom was reported in 78 patients (19.85%). PSQI score averaged 5.88 ± 3.78, and 213 patients (54.19%) were associated with the sleep disorder. The findings revealed that 67 patients had a combination of both anxiety and sleep disorders (17.04%) (Table 1).

**Table 1:** Clinical characteristics of patients with tinnitus (*n* = 393).

**Characteristics**		***N***	**%**
Gender	Male	185	47.07
	Female	208	52.93
Age (years)	≤30	36	9.20
	31–55	169	43.00
	56–79	176	44.80
	≥80	12	3.10
Tinnitus duration	<3 months	122	31.04
	3–6 months	36	9.16
	>6 months	200	50.89
	Missing	35	8.91
Localization of tinnitus	Left	118	30.03
	Right	93	23.66
	Both ears	182	46.31
Tinnitus frequency (Hz)	≤500	138	35.11
	500–3,000	32	8.14
	≥3,000	220	55.98
	Missing	3	0.76
Tinnitus loudness (dB)	≤25	48	12.21
	≥26	310	78.88
	Missing	35	8.91
Tinnitus sound types	Pure tones	174	44.27
	Compound tones	216	54.96
	Missing	3	0.76
Hearing status	Normal	198	50.38
	Impairment	191	48.60
	Missing	4	1.02
THI scores	0–16	92	23.41
	18–36	159	40.46
	38–56	118	30.03
	58–76	16	4.07
	78–100	8	2.04
PSQI scores	<5	180	45.80
	5–10	164	41.73
	11–15	44	11.20
	16–21	5	1.27
SAS scores	<50	315	80.15
	50–59	64	16.28
	60–69	8	2.04
	≥70	6	1.53

### Analysis of risk factors for sleep disorders and anxiety in tinnitus patients

Rank-sum test was used to compare the PSQI and SAS scores of different characteristics. The distribution of PSQI scores differed by gender, age, tinnitus localization, tinnitus loudness, tinnitus severity and anxiety severity. In comparison, SAS scores distribution was different in gender, tinnitus sound types, tinnitus severity and sleep disorders severity. There was no statistically significant difference between the different tinnitus durations, tinnitus frequency, and hearing status with the patients' SAS and PSQI scores (Table 2).

**Table 2:** Relationship between SAS scores, PSQI scores and different characteristics (*n* = 393).

**Characteristics**		**PSQI scores**			**SAS scores**		
		**M (IQR)**	**Z/K**	***p*-Value**	**M (IQR)**	**Z/K**	***p*-Value**
Gender	Male	5.00 (5.00)	−2.198	0.028	44.00 (6.50)	−3.326	<0.001
	Female	5.00 (6.00)			46.00 (8.00)		
Age (years)	≤30	4.50 (4.00)	11.681	0.009	44.00 (5.00)	3.453	0.327
	31–55	4.00 (5.00)			45.00 (7.00)		
	56–79	6.00 (6.00)			45.00 (7.00)		
	≥80	6.00 (3.00)			44.50 (9.75)		
Duration (months)	<3	5.00 (5.00)	0.692	0.708	45.00 (7.00)	0.049	0.976
	3–6	4.00 (6.00)			45.00 (8.00)		
	>6	5.00 (5.00)			45.00 (7.00)		
Localization	Left	4.00 (5.00)	5.673	0.059	45.00 (7.00)	0.979	0.613
	Right	5.00 (5.00)			45.00 (7.00)		
	Both	5.00 (6.00)			45.00 (8.00)		
Frequency (Hz)	≤500	5.00 (5.00)	0.782	0.676	45.00 (8.00)	0.271	0.873
	500–3,000	5.00 (7.00)			44.00 (5.75)		
	≥3,000	5.00 (5.00)			45.00 (6.00)		
Loudness (dB)	≤25	4.00 (5.00)	−1.827	0.068	44.50 (6.00)	−0.147	0.883
	≥26	5.00 (6.00)			45.00 (7.00)		
Tinnitus sound types	Pure	5.00 (5.00)	−0.212	0.832	44.00 (7.00)	−2.373	0.018
	Compound	5.00 (6.00)			45.00 (7.00)		
Hearing status	Normal	5.00 (5.00)	−1.081	0.280	45.00 (6.25)	−1.577	0.115
	Impairment	5.00 (6.00)			45.00 (9.00)		
THI	Slight	3.50 (3.00)	95.872	<0.001	40.50 (5.75)	79.940	<0.001
	Mild	4.00 (4.00)			45.00 (6.00)		
	Moderate	8.00 (5.00)			46.00 (8.00)		
	Severe	9.000 (6.00)			51.00 (7.00)		
	Catastrophic	11.00 (7.00)			66.00 (20.75)		
Sleep disorders	Normal				44.00 (6.00)	45.125	<0.001
	Mild				45.00 (8.00)		
	Moderate	-	-	-	49.00 (6.75)		
	Severe				48.00 (22.00)		
Anxiety	Normal	4.00 (4.00)	48.349	<0.001			
	Mild	8.00 (5.00)					
	Moderate	10.50 (6.00)			-	-	-
	Severe	12.50 (9.00)					

M, median; IQR, interquartile range; Z, Mann-Whitney U test statistic; K, Kruskal-Wallis H test statistic; THI, Tinnitus Handicap Inventory; SAS, Self-rating Anxiety Scale; PSQI, Pittsburgh Sleep Quality Index.

Binary logistic regression analysis was performed with anxiety or sleep disorders as independent variables to investigate the association with different characteristics. Furthermore, after FDR correction for multiple tests, the result showed that tinnitus severity and anxiety were associated with sleep disorders (FDR < 0.05). Then, the correlation factors were included in the multiple regression analysis by a standard of FDR < 0.15. We found that tinnitus severity (OR = 2.761, *p* < 0.001) and anxiety severity (OR = 3.935, *p* = 0.001) were positively associated with the occurrence of sleep disorders (Table 3).

**Table 3:** Logistic regression results for risk factors associated with sleep disorders.

**Variables**	**Simple regression analysis**				**Multiple regression analysis**		
	**B**	***p*-Value**	**OR (95%CI)**	**FDR**	**B**	***p*-Value**	**OR (95%CI)**
Gender (female)	0.359	0.331	1.432 (0.695–2.953)	0.509			
Age	0.412	0.118	1.510 (0.900–2.534)	0.262			
Duration	−0.222	0.289	0.801 (0.532–1.207)	0.487			
Localization	0.160	0.454	1.173 (0.773–1.781)	0.605			
Frequency	−0.173	0.387	0.841 (0.568–1.245)	0.553			
Loudness	1.081	0.089	2.946 (0.849–10.226)	0.223			
Tinnitus sound types	0.561	0.138	1.753 (0.835–3.681)	0.276			
Hearing loss	0.392	0.292	1.480 (0.714–3.069)	0.487			
THI	1.558	<0.001	4.751 (2.564–8.803)	<0.001	1.118	<0.001	2.761 (1.924–3.961)
SAS	1.998	0.002	7.376 (2.091–26.021)	0.010	1.370	0.001	3.935 (1.793–8.636)

B, partial regression coefficient; OR, odds ratio; CI, confidence interval; FDR, false-discovery rate corrected p-value. Variables with FDR < 0.15 were included in the multiple logistic regression equation.

The same analysis was performed to analyze factors related to anxiety. Binary logistic regression analysis revealed that gender, hearing loss, tinnitus severity, and sleep disorders were influencing factors for anxiety (FDR < 0.05). The female gender (OR = 2.526, *p* = 0.008), hearing loss (OR = 2.901, *p* = 0.003), tinnitus severity (OR = 1.863, *p* = 0.003) and sleep disorders (OR = 2.150, *p* = 0.001) were found to be significantly and positively associated with the development of anxiety as independent risk factors. An increase in sleep disorders degrees was associated with a higher risk of anxiety symptoms (Table 4).

**Table 4:** Logistic regression results for risk factors associated with anxiety.

**Variables**	**Simple regression analysis**				**Multiple regression analysis**		
	**B**	***p*-Value**	**OR (95%CI)**	**FDR**	**B**	***p*-Value**	**OR (95%CI)**
Gender (female)	1.078	0.006	4.405 (1.519–12.770)	0.024	0.927	0.008	2.526 (1.268–5.035)
Age	0.054	0.867	1.055 (0.562–1.983)	0.963			
Duration	−0.092	0.719	1.071 (0.807–1.420)	0.846			
Localization	0.014	0.958	1.014 (0.604–1.702)	0.981			
Frequency	−0.464	0.055	0.629 (0.391–1.010)	0.183			
Loudness	0.478	0.553	1.017 (1.003–1.032)	0.691			
Tinnitus sound types	0.011	0.981	1.011 (0.407–2.512)	0.981			
Hearing loss	1.099	0.033	3.000 (1.094–8.225)	0.110	1.065	0.003	2.901 (1.437–5.853)
THI	1.420	<0.001	4.138 (2.094–8.178)	<0.001	0.662	0.003	1.863 (1.243–2.792)
PSQI	1.049	0.001	2.855 (1.536–5.305)	0.007	0.920	0.001	2.510 (1.491–4.223)

B, partial regression coefficient; OR, odds ratio; CI, confidence interval; FDR, false-discovery rate corrected p-value. Characteristics with FDR < 0.15 were included in the multiple logistic regression equation.

### Relationship between tinnitus, sleep disorders and anxiety

**Figure 1:**
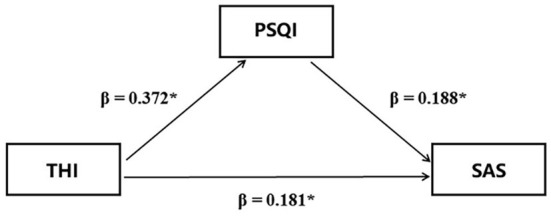
Simple mediation model for the relationship between anxiety as measured *via* the SAS and tinnitus severity as measured *via* the THI. THI, Tinnitus Handicap Inventory; SAS, Self-rating Anxiety Scale; PSQI, Pittsburgh Sleep Quality Index; β, regression coefficient; *, *p* < 0.001.

Patients' sleep disorders and anxiety are concomitant symptoms of tinnitus, and regression analysis revealed statistically significant relationships between tinnitus severity, sleep disorders, and anxiety. We utilized the mediation model to examine the extent to which this effect was direct vs. mediated by sleep disorders. The mediation analysis (*n* = 393) showed that tinnitus severity had a positive effect on anxiety [β = 0.181, *p* < 0.001, 95% CI: (0.091, 0.280)] and this model explained 21% of the variance. Tinnitus severity had a positive effect on sleep disorders [β = 0.372, *p* < 0.001, 95% CI: (0.306, 0.441)], while sleep disorders severity had a positive effect on anxiety [β = 0.188, *p* < 0.001, 95% CI: (0.084, 0.298)] as measured using the PSQI score. The mediating effect of sleep disorders severity between tinnitus severity and anxiety accounted for 27.88% [β = 0.070, *p* < 0.001, 95% CI: (0.034, 0.120)], of the total effect [β = 0.251, *p* < 0.001, 95% CI: (0.176, 0.339)], while the direct effect of tinnitus severity accounted for 72.12% (Figure 1).

## Discussion

Tinnitus may worsen patients' mental status, with chronic tinnitus patients exhibiting a series of symptoms such as poor concentration, depression, anxiety and sleep disorders (Baguley et al., 2013). Factors such as anxiety and insomnia were substantially connected with tinnitus, but the further relationship between these psychosomatic factors and tinnitus remains unclear. By assessing tinnitus characteristics, sleep quality scores and anxiety scale scores of tinnitus patients, we found that patients with higher THI levels reported higher SAS and PSQI scores. Overall, the result indicated that the risk factors for anxiety were hearing loss, female gender, sleep disorders, and tinnitus severity. Meanwhile, sleep disorders may be a mediating factor of tinnitus affecting anxiety. Our study exemplifies the clinical characteristics of tinnitus prevalence in the local region and enriches the evidence for tinnitus and psychological comorbidity.

Negative emotions associated with tinnitus can activate a stress response in the limbic system, resulting in increased sympathetic responses and dysfunction. These regions are also vital response sites for disorders such as anxiety and can indirectly influence the patient's psychological state (Pattyn et al., 2016; Richards et al., 2020). Anxiety would increase the sensitivity of patients to tinnitus, aggravate the subjective discomfort, reduce tolerance, and often lead to exaggerated tinnitus symptoms. In this study, the prevalence of anxiety was 19.85% (78 cases), and analysis revealed that women had higher SAS scores and a higher risk of anxiety than men (OR = 2.526), corroborating the previous study's findings (Craske and Stein, 2016; Fioretti et al., 2020). This phenomenon could be explained by the periodic fluctuation of female hormone levels, which affects the central emotional regulation region, making women more vulnerable to stress and thus anxiety (Solomon and Herman, 2009). We also observed differences in the occurrence of anxiety in female patients of different age groups. Patients older than 56 years had a lower risk of anxiety (OR = 0.421, *p* = 0.032), which may be attributable to the increased family and work pressures faced by younger and middle-aged women.

Hearing loss is most commonly caused by age-related hearing impairment, with age and hearing loss both considered to be associated with bothersome tinnitus (Basso et al., 2020). The cochlear basement membrane hair cells can sense high-frequency sound waves and are susceptible to genetic and environmental factors. The decrease of hair cells results in high-frequency hearing loss and may lead to tinnitus frequency predominantly above 3,000Hz (Shapiro et al., 2021). Hearing loss was also a risk factor for anxiety in tinnitus patients. It has been demonstrated that patients frequently exhibit social inhibition due to hearing impairments, which can lead to various family, social, and psychological issues, especially in cases of rapid hearing loss (Arslan et al., 2018). Our results showed a prevalence of hearing loss of 51.65%, similar to 59.1–63.6% reported by Xu et al. (2016) and Natalini et al. (2020). Patients with hearing loss were significantly more likely to experience anxiety than those without hearing loss (OR = 2.901). Anxiety levels in the hearing-impaired patients are proportional to the severity of hearing loss and decline just after surgical treatment (Shoham et al., 2019). In patients with profound bilateral deafness, cochlear implants reduce the severity of anxiety in addition to tinnitus.

Patients with tinnitus frequently report difficulty sleeping or poor sleep quality as a result of the tinnitus sound. This may be due to the fact that the quiet environment at bedtime makes tinnitus more noticeable and makes it difficult for patients to fall asleep. The deterioration of sleep patterns increases the prevalence of sleep disorders in the elderly, making it more difficult to fall or remain asleep (Gulia and Kumar, 2018). Sleep deprivation leads to the dysregulation of the circadian rhythm of cortisol and impaired executive function, impairing the ability to regulate or suppress anxiety symptoms (Chance Nicholson and Pfeiffer, 2021; Szczepek and Mazurek, 2021). As generalized anxiety disorder has both subjective sleep disturbance and sleep architecture changed, thus sleep disturbance may be one of its etiologies (Cox and Olatunji, 2016). In this study, the PSQI score was evaluated to be significantly correlated with the THI score, and the risk of sleep disorders increased with the severity level of tinnitus. Furthermore, sleep disorders can be both a cause or consequence of mental disorders such as anxiety, as anxiety may a major risk factor for the development of sleep disorders (Ohayon and Roth, 2003; LeBlanc et al., 2009; Cronlein et al., 2016).

Our study included tinnitus patients without any history or treatment of psychiatric illness and has preliminarily shown that tinnitus is strongly related to sleep disorders and anxiety. There were two possible connections: (1) Tinnitus caused anxiety and sleep disorders; (2) anxiety and sleep disorders were concomitant symptoms of tinnitus and aggravated the discomfort of tinnitus. We tested the hypothesis that sleep disorders may be a significant factor in the association between tinnitus severity and anxiety. In our study, the comorbidity rate of sleep disorders and anxiety increased with tinnitus severity, eventually exceeding 80%. As sleep disorders varied from normal to severe, the risk of anxiety increased significantly with each increased level. Additionally, sleep disorders accounted for 27.88% of the mediating effect between tinnitus severity and anxiety. Although we could not directly evaluate the causal relationship between tinnitus and psychiatric disorders, our study strongly correlated tinnitus symptoms with anxiety and sleep disorders. We further identified and highlighted sleep disorders' significant role in anxiety.

The severity of tinnitus is highly associated with depression, anxiety and neuroticism, emphasizing the importance of psychological factors in tinnitus management (Strumila et al., 2017). In patients with profound bilateral deafness, cochlear implants reduce the severity of anxiety in addition to tinnitus (Yang et al., 2021). A study in Swedish indicated that the decrease in depression symptoms is associated with a reduction in tinnitus prevalence and severity (Hebert et al., 2012). It is suggested that focusing on the treatment of sleep disorders can also be beneficial in alleviating patients' anxiety, as failure to treat tinnitus symptoms or intervene with psychosomatic problems timely may result in a vicious cycle of tinnitus-sleep disorders-anxiety (Cox and Olatunji, 2016; Chance Nicholson and Pfeiffer, 2021).

Younger patients were more likely to have anxiety in previous reports, whereas patients with tinnitus for over a year were less likely to have anxiety (Xu et al., 2016). However, no significant relationships between anxiety and characteristics such as age and duration of tinnitus were discovered in this study, which could be attributed to the uneven age distribution of the included population, which tends to be older. The intertemporal delineation of tinnitus duration in this study was different, the effect of tinnitus duration on anxiety and sleep disturbance requires further investigation.

## Limitations and conclusion

As this was a cross-sectional study with relatively small sample size, the order of variable inclusion in the mediation model represents the correlation only. The results do not allow for causal conclusions to be drawn about tinnitus, sleep disorders and anxiety. A more extensive longitudinal study is needed to determine the psychological profile and influencing mechanisms of Chinese tinnitus patients. Anxiety and sleep disorders were described briefly in this study, the relationship between different anxiety and sleep disorders components and tinnitus has not been investigated. However, the observed network of potential associations between variables suggests that future research should investigate the precise role of anxiety and sleep disturbance in tinnitus patients and the general population.

In conclusion, anxiety and sleep disorders are frequently associated with tinnitus patients, and the prominent risk factors for anxiety symptoms are female gender, hearing loss, moderate or severe tinnitus, and sleep disorders. Tinnitus can influence the occurrence of anxiety through sleep disorders, but the precise mechanism remains to be determined. Sleep management and psychological interventions are essential in the treatment of tinnitus patients. It is even more critical to focus on anxiety prevention, diagnosis, and prompt referral treatment for tinnitus patients with sleep disorders.

## Data availability statement

The raw data supporting the conclusions of this article will be made available by the authors, without undue reservation.

## Ethics statement

The studies involving human participants were reviewed and approved by the Medical Ethics Committee of the Shanghai Changzheng Hospital. The patients/participants provided their written informed consent to participate in this study.

## Author contributions

YD, CL, and HL: study conception and design. FL and TL: acquisition of data. FL, SW, and XC: analysis and interpretation of data. TW, XC, and SW: statistical analysis. SW: draft manuscript. WW, ZZ, HL, XY, and YD: revision of manuscript for important intellectual content. All authors contributed to the article and approved the submitted version.

## Funding

This work was supported by a grant (2019-QH-24) from 2019 Military Medicine Special Fund, Naval Medical University (Second Military Medical University) Shanghai, China, and a grant from the National Natural Science Foundation of China (No. 81870702).

## Conflict of interest

The authors declare that the research was conducted in the absence of any commercial or financial relationships that could be construed as a potential conflict of interest.

## Publisher's note

All claims expressed in this article are solely those of the authors and do not necessarily represent those of their affiliated organizations, or those of the publisher, the editors and the reviewers. Any product that may be evaluated in this article, or claim that may be made by its manufacturer, is not guaranteed or endorsed by the publisher.

## Abbreviations

THI: Tinnitus Handicap Inventory
SAS: Self-rating Anxiety Scale
PSQI: Pittsburgh Sleep Quality Index
N: number
M: median
IQR: interquartile range
SD: standard deviation
Z: Mann-Whitney U test statistic
K: Kruskal-Wallis H test statistic
B: partial regression coefficient
OR: odds ratio
CI: confidence interval.
